# Great Apes' Risk-Taking Strategies in a Decision Making Task

**DOI:** 10.1371/journal.pone.0028801

**Published:** 2011-12-21

**Authors:** Daniel B. M. Haun, Christian Nawroth, Josep Call

**Affiliations:** 1 Max Planck Research Group for Comparative Cognitive Anthropology, Max Planck Institute for Evolutionary Anthropology, Leipzig, Germany; 2 Max Planck Research Group for Comparative Cognitive Anthropology, Max Planck Institute for Psycholinguistics, Nijmegen, The Netherlands; 3 Department of Psychology, University of Portsmouth, Portsmouth, United Kingdom; 4 Department for Developmental and Comparative Psychology, Max Planck Institute for Evolutionary Anthropology, Leipzig, Germany; Yale University, United States of America

## Abstract

We investigate decision-making behaviour in all four non-human great ape species. Apes chose between a safe and a risky option across trials of varying expected values. All species chose the safe option more often with decreasing probability of success. While all species were risk-seeking, orangutans and chimpanzees chose the risky option more often than gorillas and bonobos. Hence all four species' preferences were ordered in a manner consistent with normative dictates of expected value, but varied predictably in their willingness to take risks.

## Introduction

Everyday, we face situations requiring us to decide between options for which we weigh how much we might get out of it and how likely we are to get it if we tried. Any agent, in order to successfully navigate a world of possibilities, needs to strike the right balance between these factors, utilizing mechanisms that when confronted with risky choices, lead to decisions, which optimally combine the probability of receiving a reward multiplied by the amount of the reward (expected value: EV). Additionally to the ability to make weighed choices in risky situations, individuals might have certain preferences when negotiating risk, even if all available options have identical expected values. For example, in scenarios with stable expected values, but varying levels of risk humans tend to choose the safe over the risky option. When for example asked to chose between a box containing 10 Euros for certain or another box with a 50/50 chance of containing 20 Euros or being empty, human subjects prefer the safe option [1].

Non-human animals largely appear to share the human preference to avoid risk. A comparison of risk sensitivity across a large number of species found most to be either risk averse or risk neutral. Risk seeking species appear to be rare [2]. Given this background, comparisons across the primate family have documented a surprising amount of variation both between [2] and within species depending on the task [3]. While some species, for example bonobos, appear risk averse, other species, for example chimpanzees prefer risky over safe options [4]. There have been a number of recent attempts to elucidate the socio-ecological determinants of such inter-specific variability using the comparative method [5], [6]. Thus, feeding ecology has been associated both with risk preference in chimpanzees and bonobos [4] and delay of gratification in callitrichids [7]. Similarly, socio-ecological factors have also been linked with certain cognitive abilities. For example, fission-fusion dynamics have been associated with inhibitory control in primates [8] and social complexity has been linked to transitive inference and behavioral flexibility in corvids [9]. Here we investigate decision-making in four members of the great ape family: Orangutans (*Pongo abelii*), gorillas (*Gorilla gorilla*), bonobos (*Pan paniscus*), and chimpanzees (*Pan troglodytes*). Our aim is to extend what is known from prior research in three ways. First, we test whether non-human great apes choose based on expected value in a risky scenario (optimally combining probability of success and the value of the reward) by varying both the probability of success and the relative value of the risky vs. the safe choice. Second, we test whether non-human great apes consider their own level of uncertainty when choosing options in a risky scenario by varying the amount of relevant information available to the individual. Third, we aim to validate prior results documenting differences in risk-preferences between chimpanzees and bonobos [4] with a different task and extend the number of great ape species in order to evaluate different explanations for the interspecific variation in risk preference.

## Methods

### Subjects

We tested eight chimpanzees, five bonobos, six orangutans, and three gorillas between the age of 4 and 35 years (see Table S1). There were eight males and 14 females. All apes were born in captivity and were housed in social groups at the Wolfgang Köhler Primate Research Center (WKPRC) in Zoo Leipzig (Germany). The apes were housed in semi-natural indoor and outdoor enclosures with a minimum size of 2000 m^2^ per species, regular feeding schedules, enrichment, and water ad lib. Rewards were highly valued food-items. Prior to this study all apes had participated in various studies on social and physical cognition. Apes were neither food nor water deprived during testing or at any other time.

### Apparatus

**Figure 1:**
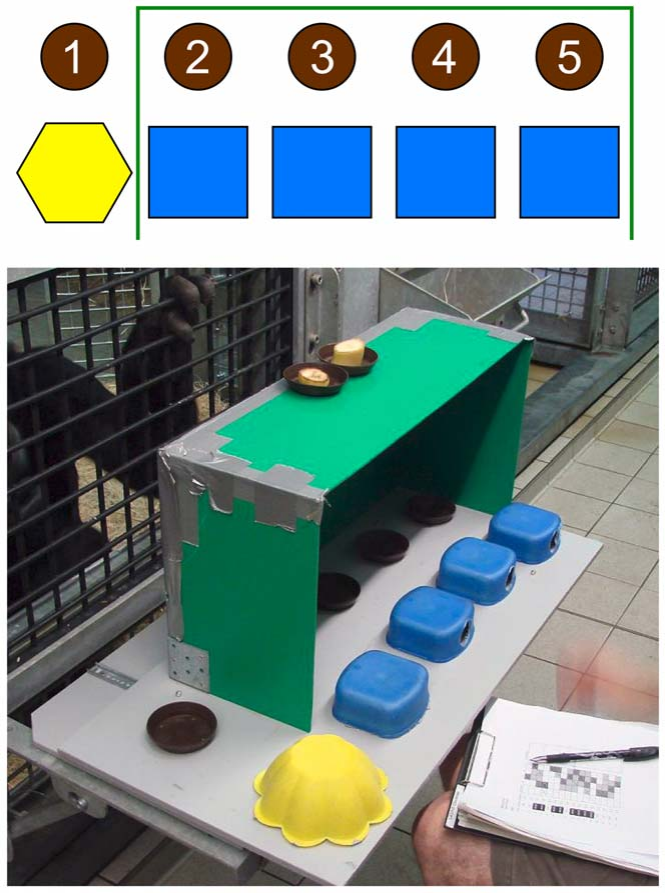
Experimental setup faced by the subjects. Depicted are the safe cup (yellow) the risky cups (blue) and bowls (brown) in addition to the barrier (green) blocking visual access to the blue cups during the baiting in hidden trials.

Five small brown bowls forming a straight line were placed on a table that could be slid forward within the subject's reach. Additional materials included: a large hexagonal yellow cup (cup 1), 4 smaller identical square blue cups (cups 2–5), and a barrier to block visual access to the blue cups. On top of the visual occluder two additional brown bowls were placed left and right of the midline (see Figure 1).

### Procedure

All procedures were non-invasive and subjects could choose to stop participating at any time. Animal husbandry and research complied with the “EAZA Minimum Standards for the Accommodation and Care of Animals in Zoos and Aquaria”, the “EEP Bonobo Husbandry Manual” for the Bonobo group in particular, the “EAZA Code of Practice Article 4: Research” and the “WAZA Ethical Guidelines for the Conduct of Research on Animals by Zoos and Aquariums”.

On every trial subjects faced a table with five small, empty bowls placed in a row. The experimenter then placed two pieces of banana, one large one small, standing upright on the table. After approximately 2 seconds, the small piece was always placed in the bowl to the far right of the subject providing a spatially stable *safe option*. The large piece was placed in any of the remaining four brown bowls (*risky option*). Depending on the intended level of uncertainty (see below) subjects were able to witness (or not) the food's final destination. The small piece was always hidden under a yellow cover providing a stable colour cue identifying the safe option additionally to the stable spatial location. The large piece was always hidden under a blue cover.

Choosing based on EV: In order to test subjects' abilities to choose based on EV, we varied both the probability of success and the relative value of the risky option. Placing an additional number of blue cups over some or all of the remaining empty brown bowls varied the probability of success. As a result a random choice amongst blue cups would result in the following probabilities (P): 1 blue cup: P = 1; 2 blue cups: P = .5; 3 blue cups: P = .33; 4 blue cups: P = .25. Decreasing the size of the small piece of banana varied the relative value (V) of the large piece (always 3 cm in size) over the small piece in three steps: small piece = 0.5 cm: V = 6; small piece = 1.0 cm: V = 3; small piece = 2.0 cm: V = 1.5. Table 1 specifies the expected values (EV = P×V) across different relative values (V) and probabilities of success (P). If non-human great apes decide based on EV in risky situations, considering both, relative value and probability of success, they should chose the risky option more often in scenarios with higher EV than in scenarios with a low EV.

**Table 1:** Expected values across different combinations of relative value of the large reward over the small reward (V) and probabilities of success (P).

	(V)		
(P)	1.5	3	6
.25	.375	.75	1.5
.33	.5	1	2
.5	.75	1.5	3
1	1.5	3	6

In order to test whether non-human great apes consider their own level of uncertainty we hid the risky option either in full view of the subject (visible condition) or hidden behind an occluder (hidden condition). In the visible condition, subjects witnessed under which blue cup the large piece was hidden. Therefore, in all visible trials, the probability to find the large piece, if the subjects could remember the location correctly, was P = 1. In hidden trials, the probability of success varied between .25 and 1 from trial to trial (see Table 1). If non-human great apes considered their own level of uncertainty when making choices in risky situations, they should choose the risky option more often on visible than hidden trials. This prediction is based on data from previous studies showing that apes select the riskier option more often in visible than hidden trials [10]–[12]. Once all cups were placed (and the occluder was removed on the hidden trials) the ape could choose one of the available options by touching one of the cups. In response the experimenter handed subjects whatever was under the cup they had indicated. In the case the cup was empty, the experimenter opened all cups and removed the remaining food.

We administered six 16-trial sessions. Two consecutive sessions used one particular size piece of banana as the safe option. The order in which the different size safe options were administered was counterbalanced across subjects. Within each session, probability of success (.25, .33, .5, 1), the order in which safe and risky options were hidden (safe first, risky first) and the uncertainty of the subjects (visible, hidden) were pseudo-randomized across trials.

Prior to presenting a new safe option size, we administered a pre-test to assess whether subjects could discriminate between the larger (risky option) and the smaller (safe option) reward sizes. Both rewards were placed standing up in the middle of the table. After 2 seconds, the two pieces were placed in the two outmost brown bowls and covered by two blue inverted cups. The location and the order of food placement were counterbalanced across trials. Each subject had to choose the larger reward on 4 consecutive trials in order to pass the pre-test.

### Coding and data analysis

All sessions were videotaped and subjects' choices were coded live during the test. Reliability was assessed on 15% of trials (randomly chosen) and was almost perfect (Cohen's kappa = 0.97). Since some of the data did not meet the normality assumption, we used non-parametric statistics throughout. We analyzed effects of species, visibility (hidden / visible), the relative size of the safe reward (1/6, 1/3, 2/3) and the number of risky cups (1–4) on the probability of choosing the risky option. We also analysed whether EV (see Table 1) was a good predictor of subjects' risky choices by applying a linear, quadratic, logarithmic, and inverse function for visible and hidden trials. We reported the function that produced the best fit. We were particularly interested to see whether subjects would change their choice tendencies at transition points when the expected value is 1 (i.e., the relative size of the safe to the risky option equals the probability of success for the risky option). We expected subjects to choose the safe rather than the risky option when EV<1 but select the risky rather than the safe option when EV>1 (see Table 1). Furthermore, we tested whether the four species would vary in their risk preferences as suggested by earlier work on chimpanzees and bonobos [4] by comparing the percentage of risky choices across species.

Finally, we conducted two auxiliary (control) analyses. One analysis assessed the possibility that subjects changed their choices with repeated testing. We did this in two ways. First, we compared the percentage of choices directed at the risky option in the first and second session of each of the three sizes of the safe reward pooling visible and hidden trials. Second, we compared the first and second half of the trials in the first session for each of the three sizes of the safe reward. Analyzing the responses of hidden trials only produced the same result. The second auxiliary analysis assessed subjects' accuracy for selecting the baited cup from the risky alternative as a function of the size of the safe reward and the number of cups available in the risky alternative. This analysis allowed us to assess whether subjects had paid attention to the location of the food in visible trials and whether they were using inadvertent cues to locate the baited cup in hidden trials.

## Results

**Figure 2:**
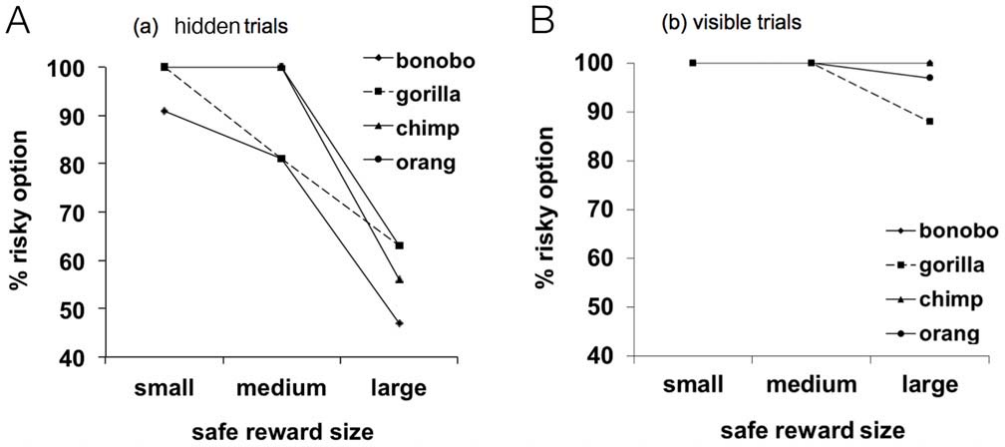
Mean % of trials in which subjects selected the risky option as a function of species and size of the safe reward for (a) hidden and (b) visible trials.

Subjects overall chose the risky option more often in the visible than hidden trials (Wilcoxon test: Z = 3.73, P<0.001; visible: mean = 98.1, SEM = 0.6; hidden: mean = 83.7, SEM = 2.2). Furthermore, choosing the risky option decreased as the size of the safe option became larger (Friedman test: χ^2^ = 25.51, df = 2, P<0.001, see Figure 2). Although this effect was present in both visible (Friedman test: χ^2^ = 19.0, df = 2, P<0.001) and hidden trials (Friedman test: χ^2^ = 25.72, df = 2, P<0.001), the curve was steeper in the latter compared to the former. Subjects also chose the risky option less often when confronted with a larger number of cups (Friedman test: χ^2^ = 9.24, df = 3, P = 0.026). This result, however, could not be confirmed for hidden (Friedman test: χ^2^ = 5.86, df = 3, P = 0.118) or visible trials separately (Friedman test: χ^2^ = 3.52, df = 3, P = 0.32).

**Figure 3:**
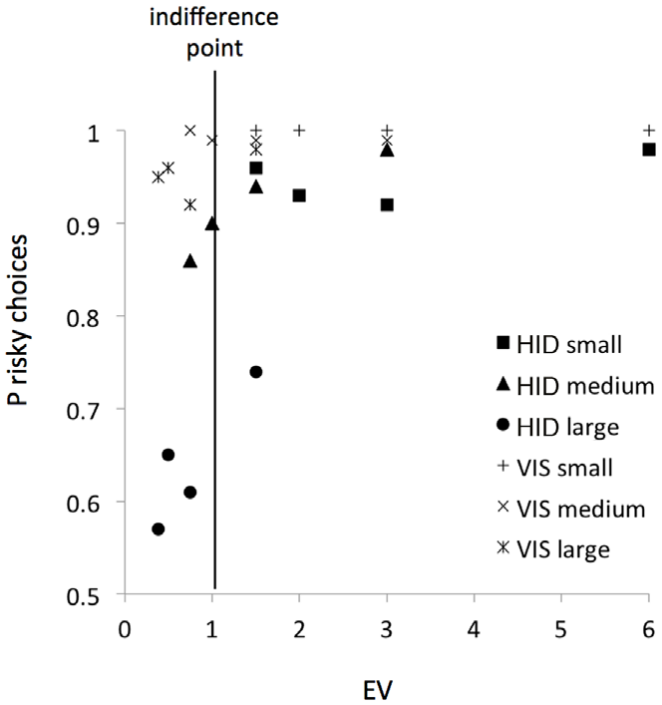
Probability to select the risky option as a function of EV for (a) hidden and (b) visible trials. Also depicted are the values corresponding to each of the sizes of the safe reward.

Next we analysed how accurately the EV predicted subjects' choices in the visible and hidden trials (Figure 3). We used the EV scores displayed in Table 1 and correlated them with the corresponding choices of the risky option in each of the 12 possible cells. There was a significant correlation between EV and choices both for visible (Spearman r = 0.65, P = 0.023, N = 12) and hidden trials (Spearman r = 0.85, P<0.001, N = 12). In both cases, inverse functions produced the best fit of the choice data (visible: R^2^ = 0.37, F = 5.98, df = 10, P = 0.034, Y = 1.001−(0.021/EV); hidden: R^2^ = 0.71, F = 23.98, df = 10, P = 0.001, Y = 1.001−(0.019/EV); Figure 3). Also in both cases, subjects showed a strong tendency to select the risky option. Note that EV = 1 represents the point of indifference between the safe and the risky options and if subjects were solely choosing based on this parameter, they should be selecting at around 50%. Clearly, this was not the case (see Figure 3). Although the high percentage of risky choices in visible trials is understandable since subjects knew where the food was located, this was not the case for hidden trials with more than one cup.

Focusing on hidden trials (Figure 2a), species did not significantly differ in the percentage of trials on which they chose the risky option with the small (Kruskal-Wallis test: χ^2^ = 1.62, df = 3, P = 0.66) or the large size safe options (Kruskal-Wallis test: χ^2^ = 3.53, df = 3, P = 0.32). In contrast, there were species differences with the medium size safe option (Kruskal-Wallis test: χ^2^ = 11.17, df = 3, P = 0.011). Post-hoc tests indicated that bonobos chose the risky option significantly less often than chimpanzees (Mann-Whitney exact test: U = 2.5, P = 0.024) and orangutans (Mann-Whitney exact test: U = 0.0, P = 0.01). Moreover, gorillas also chose the risky option less often than orangutans (Mann-Whitney test: Z = 2.12, P = 0.034). In contrast, there were no species differences in visible trials for any of the three sizes of the safe option (Kruskal-Wallis tests: small: χ^2^ = 0, df = 3, P = 1.0; medium: χ^2^ = 3.00, df = 3, P = 0.39; large: χ^2^ = 6.38, df = 3, P = 0.095).

Analysing the hidden trials in more detail within each combination of relative value and probability of success confirmed this result (Table 2). There were no significant differences between species for the small size safe option, regardless of the probability of success (Kruskal-Wallis test: χ^2^<6.08, df = 3, P>0.10 in all cases). Similarly, there were no significant differences between species for the large size safe option regardless of the probability of success (Kruskal-Wallis test: χ^2^<5.12, df = 3, P>0.16 in all cases). In contrast, species significantly differed in the medium size safe option in some scenarios (Kruskal-Wallis test: 1-cup: χ^2^ = 11.96, df = 3, P = 0.008; 2-cup: χ^2^ = 6.21, df = 3, P = 0.102; 3-cup: χ^2^ = 7.50, df = 3, P = 0.058; 4-cup: χ^2^ = 7.86, df = 3, P = 0.049). However, post-hoc test failed to reveal any significant inter-specific differences (Mann-Whitney exact test: P>0.066 in all cases).

**Table 2:** Median % of trials directed at the risky option as a function of species, safe reward size, and number of risky cups available.

	safe reward size											
	small				medium				large			
species	1	2	3	4	1	2	3	4	1	2	3	4
bonobo	100	75	100	100	100	87.5	75	50	62.5	50	37.5	50
gorilla	100	100	100	100	75	75	75	100	75	75	75	50
chimpanzee	100	100	100	100	100	100	100	100	100	50	75	62.5
orangutan	100	100	100	100	100	100	100	100	75	62.5	50	75
all species	100	100	100	100	100	100	100	100	75	50	75	50

To assess potential learning effects, we compared the first and second session for each size of the safe reward but found no change across sessions (Wilcoxon tests: Small: z = 0.09, P = 0.93; 1^st^ session: mean = 97.3, SEM = 1.2; 2^nd^ session: mean = 97.3, SEM = 1.3; Medium: z = 0.51, P = 0.61; 1^st^ session: mean = 95.9, SEM = 1.9; 2^nd^ session: mean = 95.0, SEM = 1.8; Large: z = 0.39, P = 0.70; 1^st^ session: mean = 80.1, SEM = 2.6; 2^nd^ session: mean = 79.5, SEM = 2.9 ). Similarly, we found no evidence that subjects changed their choices when comparing the first half with the second half of the first session (Wilcoxon tests: Small: z = 1.13, P = 0.26; 1^st^ half: mean = 98.2, SEM = 1.3; 2^nd^ half: mean = 96.4, SEM = 1.5; Medium: z = 0.45, P = 0.66; 1^st^ half: mean = 96.3, SEM = 2.0; 2^nd^ half: mean = 95.6, SEM = 2.1; Large: z = 0.46, P = 0.65; 1^st^ half: mean = 78.6, SEM = 3.7; 2^nd^ half: mean = 81.6, SEM = 3.8).

Finally, there were no significant differences in the likelihood of finding the reward from the risky cups (i.e., retrieval accuracy) as a function of the size of the safe reward in visible (Friedman test: χ^2^ = 4.20, df = 2, P = 0.122) or hidden trials (Friedman test: χ^2^ = 1.37, df = 2, P = 0.504). Similarly, there were no differences in retrieval accuracy as a function of the number of cups (we excluded trials with just one cup because subjects could not be wrong) in visible trials (Friedman test: χ^2^ = 3.47, df = 2, P = 0.177). In contrast, and unsurprisingly, subjects' retrieval accuracy in the hidden condition decreased as the number of cups available increased (Friedman test: χ^2^ = 17.88, df = 2, P<0.001; 2 cups: mean = 47.0, SEM = 3.2; 3 cups: mean = 31.4, SEM = 2.8; 4 cups: mean = 23.5, SEM = 3.0).

## Discussion

As predicted, subjects chose the safe option more often when they had not witnessed the reward being placed under one of the blue cups, indicating sensitivity for their own level of uncertainty [10], [11], [13]. Additionally, safe choices increased with the size of the safe option relative to the risky option and with a decrease in the probability of success for the risky option. Taken together, this means that subjects' choices were regulated by multiple factors including their own uncertainty, the relative value of rewards, and the probability of success. This is even more remarkable considering that there was no evidence that subjects learned to produce those responses during the course of the experiment. This is important because learned contingencies are one of the main explanations invoked in experiments investigating decision making in uncertain situations [14].

Our results confirmed the difference between chimpanzees and bonobos in risk sensitivity [4] with a different method. Additionally, our results showed that orangutans, just like chimpanzees, were also risk-prone whereas gorillas tended to be more conservative but not as much as bonobos. It is often assumed that chimpanzees' risk proneness is correlated with potentially costly, risky strategies like coordinated hunting [15] and particularly extensive tool use [16]–[18]. Compared with the similarly risk-seeking orangutans, which do not hunt and use tools less often [19], [20] this explanation is therefore less applicable for our results.

Heilbronner and colleagues [4] proposed that differences in the natural ecology of the species might explain some of the differences in risk preferences. While all ape species eat ripe fruit when it is available, chimpanzees are ripe fruit specialists [21], which means that in times of low fruit abundance they continue searching for fruit. In contrast bonobos under these circumstances switch to terrestrial herbaceous vegetation (THV), a highly consistent food source. Their preferred food continues to be ripe fruit, but they appear to have shifted to consuming higher levels of leaves, young shoots, and stem tips of high quality THV than chimpanzees [22]. The fruit consumption of lowland gorillas is somewhat lower than that of the other apes however their intake on THV is higher than that of chimpanzees and more similar to that of bonobos [23].

Compared to Africa, Southeast Asia is subject to dramatic shifts in food availability due the periodic mast fruiting of certain trees, which affects many species including orangutans. In times of extreme fruit scarcity (immediately following a super-abundant mast fruiting) orangutans were observed to feed on bark [24], [25]. During non-mast years with less fluctuating fruit abundance orangutans move widely in search of ripe fruit and seeds [23]. So orangutans present a more complicated picture. However given that they just feed on bark in extreme environmental conditions and in more normal circumstances will travel long ways for fruits, one can assume that their feeding ecology is in some aspects most similar to that of chimpanzees. These similarities might explain why chimpanzees and orangutans appear to be more risk prone on foraging tasks than gorillas and bonobos. Interestingly, all tested individuals live in a zoo with highly regulated feeding schedules. This means that differences in risk preferences based on diet would have to rely on innate predispositions due to selection for the natural foraging ecology of the species.

In contrast to Heilbronner and colleagues [4], we found an overall high rate of risky choices. This is surprising because even with a large safe option and a low probability for success, subjects never chose the risky option less than 50% of the time in any condition, resulting in a sub-optimal overall pattern of choices, even though EV predicted choices with remarkable yet not perfect accuracy. This bias towards the risky option could be explained, for instance, by a failure to inhibit a subject's inherent tendency to choose the large reward. Several studies have shown that great apes (and other primates) need a large number of trials to overcome their initial tendency to choose a higher valued food [26]–[29], even when the reward is no longer visible [29], like it was the case in our experiment. However, alternative explanations for the high risk taking, like an inadequate ability to infer the chances of the risky option without experience and therefore being biased towards a risky choice, should be taken into consideration as well.

Our high rates of risky choices are also surprising in comparison to humans, who are known to be generally risk averse for gains and risk seeking for losses [30]. Future studies should extend our experimental setup to human participants, especially children, to see how they would perform in the same task. Furthermore, great apes' should be tested on their risk preferences when negotiating losses instead of gains to create another line of comparison between humans and the other great apes. Based on our findings, we propose that decision-making in the great apes provides a promising context for the interpretation of decision-making in humans, the fifth great ape species. Finally, more primate and non-primate species need to be tested in the current and other paradigms since the inferential strength of the comparative analysis heavily relies on the number (and diversity) of species entered into the analysis.

## Supporting Information

Table S1 Overview of subjects. Information about name, sex (f = female; m = male), date of birth and rearing history are displayed.
(DOC)

## References

[1] Kahneman D, Tversky A (1979). Prospect Theory: An Analysis of Decision under Risk.. Econometrica.

[2] Kacelnik A, Bateson M (1996). Risky Theories—The Effects of Variance on Foraging Decisions.. American Zoologist.

[3] Hayden BY, Platt ML (2007). Temporal Discounting Predicts Risk Sensitivity in Rhesus Macaques.. Current Biology.

[4] Heilbronner SR, Rosati AG, Stevens JR, Hare B, Hauser MD (2008). A fruit in the hand or two in the bush? Divergent risk preferences in chimpanzees and bonobos.. Biology Letters.

[5] Haun DB, Jordan FM, Vallortigara G, Clayton NS (2010). Origins of spatial, temporal and numerical cognition: Insights from comparative psychology.. Trends Cogn Sci.

[6] MacLean E, Matthews L, Hare B, Nunn C, Anderson R (2011). How does cognition evolve? Phylogenetic comparative psychology.. Animal Cognition.

[7] Stevens JR, Hallinan EV, Hauser MD (2005). The ecology and evolution of patience in two New World monkeys.. Biology Letters.

[8] Amici F, Aureli F, Call J (2008). Fission-Fusion Dynamics, Behavioral Flexibility, and Inhibitory Control in Primates.. Current Biology.

[9] Bond AB, Kamil AC, Balda RP (2003). Social complexity and transitive inference in corvids.. Animal Behaviour.

[10] Call J, Carpenter M (2001). Do apes and children know what they have seen?. Animal Cognition.

[11] Suda-King C (2008). Do orangutans (Pongo pygmaeus) know when they do not remember?. Animal Cognition.

[12] Call J (2010). Do apes know that they could be wrong?. Animal Cognition.

[13] Hampton RR, Zivin A, Murray EA (2004). Rhesus monkeys (Macaca mulatta) discriminate between knowing and not knowing and collect information as needed before acting.. Animal Cognition.

[14] Smith JD (2009). The study of animal metacognition.. Trends Cogn Sci.

[15] Gilby IC, Wrangham RW (2007). Risk-prone hunting by chimpanzees (Pan troglodytes schweinfurthii) increases during periods of high diet quality.. Behavioral Ecology and Sociobiology.

[16] Sumita K, Kitahara-Frisch J, Norikoshi K (1985). The acquisition of stone-tool use in captive chimpanzees.. Primates.

[17] Inoue-Nakamura N, Matsuzawa T (1997). Development of stone tool use by wild chimpanzees (< xh: i> Pan troglodytes</xh: i>).. Journal of Comparative Psychology.

[18] Nishimura T, Okayasu N, Hamada Y, Yamagiwa J (2003). A case report of a novel type of stick use by wild chimpanzees.. Primates.

[19] Van Schaik CP, Knott CD (2001). Geographic variation in tool use on Neesia fruits in orangutans.. American Journal of Physical Anthropology.

[20] Fox EA, van Schaik CP, Sitompul A, Wright DN (2004). Intra-and interpopulational differences in orangutan (Pongo pygmaeus) activity and diet: Implications for the invention of tool use.. American Journal of Physical Anthropology.

[21] Wrangham RW, Conklin-Brittain NL, Hunt KD (1998). Dietary response of chimpanzees and cercopithecines to seasonal variation in fruit abundance. I. Antifeedants.. International Journal of Primatology.

[22] Wrangham RW, Chapman CA, Clark-Arcadi AP, Isabirye-Basuta G (1996). Great ape societies..

[23] Conklin-Brittain NL, Knott CD, Wrangham RW (2001). The feeding ecology of apes.. Conference Proceedings. The Apes: Challenges for the 21st Century.

[24] Suzuki A (1994).

[25] Knott C (1999).

[26] Boysen ST, Berntson GG (1995). Responses to Quantity: Perceptual Versus Cognitive Mechanisms in Chimpanzees (Pan Troglodytes).. Journal of Experimental Psychology: Animal Behavior Processes.

[27] Murray EA, Kralik JD, Wise SP (2005). Learning to inhibit prepotent responses: successful performance by rhesus macaques, Macaca mulatta, on the reversed-contingency task.. Animal Behaviour.

[28] Uher J, Call J (2008). How the Great Apes (Pan troglodytes, Pongo pygmaeus, Pan paniscus, Gorilla gorilla) Perform on the Reversed Reward Contingency Task II: Transfer to New Quantities, Long-Term Retention, and the Impact of Quantity Ratios.. Journal of Comparative Psychology.

[29] Vlamings PHJM, Uher J, Call J (2006). How the great apes (Pan troglodytes, Pongo pygmaeus, Pan paniscus, and Gorilla gorilla) perform on the reversed contingency task: The effects of food quantity and food visibility.. Journal of Experimental Psychology: Animal Behavior Processes.

[30] Kahneman D, Tversky A (2000). Choices, values, and frames.

