# Supplementary material for: Great Apes' Risk-Taking Strategies in a Decision Making Task
Source: PLoS One. 2011 Dec 21;6(12):e28801. doi: 10.1371/journal.pone.0028801 (PMC3244423; doi:10.1371/journal.pone.0028801)
Supplement: Table S1 — Overview of subjects. Information about name, sex (f = female; m = male), date of birth and rearing history are displayed. (DOC) [file pone.0028801.s001.doc]

Electronic Supplemental Material for:

**Great apes’ risk-taking strategies in a decision making task**

Daniel B.M. Haun, Christian Nawroth & Josep Call

**Table S1.** Overview of subjects. Information about name, sex (f = female; m = male), date of birth and rearing history are displayed.

| **Name** | **Sex** | **Date of birth** | **Rearing** |
| --- | --- | --- | --- |
|  |  |  |  |
| **Chimpanzees** |  |  |  |
| Fifi | f | May. 12, 1993 | parent |
| Frodo | m | Nov. 28, 1993 | parent |
| Gertrudia | f | May. 20, 1993 | parent |
| Jahaga | f | Jan. 06, 1993 | parent |
| Patrick | m | Jun. 8,1997 | parent |
| Pia | f | Sep. 16, 1999 | parent |
| Sandra | f | Jun. 9, 1993 | parent |
| Unyoro | m | Feb. 25, 1997 | parent |
|  |  |  |  |
| **Bonobos** |  |  |  |
| Joey | m | Dec. 13, 1982 | hand reared |
| Kuno | m | Nov. 26, 1996 | hand reared |
| Limbuko | m | Oct. 04, 1995 | hand reared |
| Ulindi | f | Oct. 10, 1993 | parent |
| Yasa | f | Aug. 27, 1997 |  |
|  |  |  |  |
| **Gorillas** |  |  |  |
| Gorgo | m | Jun. 28,1981 | hand reared |
| Kibara | f | Jan.13,2004 | parent |
| Viringika | f | Mar. 23,1995 | parent |
|  |  |  |  |
| **Orangutans** |  |  |  |
| Bimbo | m | Sep.20,1980 | hand reared |
| Dokana | f | Jan.31,1989 | parent |
| Dunja | f | Apr. 19,1973 | parent |
| Kila | f | Jun. 02,2000 | parent |
| Padana | f | Nov. 18, 1997 | parent |
| Pini | f | Jun.30, 1988 | parent |
